# The rickettsia: an emerging group of pathogens in fish.

**DOI:** 10.3201/eid0302.970206

**Published:** 1997

**Authors:** J L Fryer, M J Mauel

**Affiliations:** Department of Microbiology, Oregon State University, Corvallis 97331-3804, USA. fryerj@bcc.orst.edu

## Abstract

Piscirickettsia salmonis is the first of the previously unrecognized rickettsial pathogens of fish to be fully characterized. Since the recognition of P. salmonis in 1989, the impact of rickettsial pathogens in fish has become increasingly apparent. Growing awareness of the emergence of these fastidious intracellular organisms has led to the discovery of rickettsial diseases among diverse species of fish from different geographic locations and aquatic environments. The source, reservoir, and mode of transmission of these agents as well as appropriate methods of disease prevention and control remain to be established.


### The Rickettsia: an Emerging Group of Pathogens in Fish

**John L. Fryer and Michael J. Mauel**
Oregon State University, Corvallis, Oregon, USA

*Piscirickettsia salmonis* is the first of the previously unrecognized rickettsial pathogens of fish to be fully characterized. Since the recognition of *P. salmonis* in 1989, the impact of rickettsial pathogens in fish has become increasingly apparent. Growing awareness of the emergence of these fastidious intracellular organisms has led to the discovery of rickettsial diseases among diverse species of fish from different geographic locations and aquatic environments. The source, reservoir, and mode of transmission of these agents as well as appropriate methods of disease prevention and control remain to be established.

### The Emergence of Rickettsial Diseases in Fish

The earliest report of a rickettsialike organism in fish occurred in 1939 during examination of diseased *Tetrodon fahaka* from the Nile River in Egypt. The microorganisms were observed within stained tissue cells, but no attempts were made to culture them (1). In 1975 Ozel and Schwanz-Pfitzner (2) first cultured rickettsialike organisms from fish while examining rainbow trout (*Oncorhynchus mykiss*) for Egtved virus. While cultivating the virus in RTG-2 cells (3), they observed that cells also contained an intracellular rickettsialike organism. However, research on the organism was limited, and its taxonomic position and relevance to disease were not determined. The microorganism was not maintained and is no longer available for evaluation. No further reports of rickettsia in fish appeared until 1986 when Davies observed rickettsialike organisms in the tissue of dragonets (*Callionymus lyra L.*) collected in waters off the coast of Wales (4) (Table 1).

The role of rickettsiae as emerging pathogens of fish became apparent in 1989 (5), when a previously unrecognized bacterium (6) was isolated in the chinook salmon (*Oncorhynchus tshawytscha*) embryo cell line, CHSE-214 (7) and was demonstrated to be the cause of epizootics in marine-netpen-reared coho salmon species and (*Oncorhynchus kisutch*) in Region X, Chile (8).

Address for correspondence: John L. Fryer, 220 Nash Hall, Department of Microbiology, Oregon State University, Corvallis, OR 97331-3804, USA; fax: 541-737-2166; e-mail: fryerj@bcc.orst.edu.

Table 1. Unidentified rickettsialike organisms observed in and/or isolated from fish

| Fish host species | Geo. location | Salt/Fresh Water | Obs./ Iso. | Ref. |
|---|---|---|---|---|
| Fokaka (*Tetrodon fahaka*) | Egypt | F[a] | O[c] | 1 |
| Rainbow trout (*Oncorhynchus mykiss*) | Europe | F | I[d] | 2 |
| Dragonet (*Callionymus lyra*) | Wales | S[b] | O | 5 |
| Masu salmon (*Oncorhynchus masou*)[e] | Chile | S | O | |
| Mozambique tilapia (*Oreochromis mossambicus*) Nile tilapia (*O. niloticus*) Blue tilapia (*O. aureus*) Redbelly tilapia (*Tilapia zillii*) Wami tilapia (*T. hornorum*) | Taiwan | S & F | I | 15 |
| Blue-eyed plecostomus (*Panaque suttoni*) | Colombia | F | O | 16 |
| Atlantic salmon (*Salmo salar*) | Chile | F | I | 19 |
| Sea bass (*Dicentrarchus labrax*) | France | S | O | 17 |

[a]F = Host collected from fresh water
[b]S = Host collected from salt water
[c]O = Observed only
[d]I = Isolated in cell culture
[e]Sandra Bravo, Puerto Montt, Chile

*Piscirickettsia salmonis*, was the first rickettsialike organism isolated from fish, characterized, and demonstrated to cause disease in experimentally infected hosts (6). Initially, rickettsial infections of fish were thought to be confined to coho salmon in southern Chile, but the agent and the disease it causes have since been observed in other locations (Table 2). In British Columbia, Brocklebank et al. (9,10) observed that sea-farmed Atlantic salmon (*Salmo salar*) also manifested pathologic features indistinguishable from those of salmonids in Chile. The causation of the disease was established by experimental infection followed by isolation in CHSE-214 cells. The organism was morphologically similar to *P. salmonis,* but comparatively less virulent for salmonids. These researchers also provided anecdotal reports that the same condition occurred as early as 1970 in pink salmon (*Oncorhynchus gorbuscha*) held in sea water tanks in British Columbia; beginning in the 1980s, the condition was also seen in coho and chinook salmon from local sea farms rearing salmonids. Rickettsiae morphologically similar to those implicated in the diseases in Chile and Canada also caused low level mortality in Atlantic salmon held in sea water cages in Norway (11) and Ireland (12). We have identified all of these agents as *P. salmonis* by direct fluorescence antibody tests using the polyclonal antiserum (unpub. data; 13).

Until 1994, all recognized rickettsial diseases of fish had been observed in species of salmonids cultured in sea water. However, it is now apparent that these pathogens affect fish over a broad host and geographic range and in both fresh water and marine environments. Chern and Chao (14) reported an epizootic rickettsial disease in several species of tilapia in both marine and fresh water ponds in Taiwan (Table 1). Death rates associated with this disease reached 95% at certain sites. Khoo et al. (15) reported a rickettsialike organism in moribund specimens of a fresh water tropical fish, the blue-eyed plecostomus (*Panaque suttoni*), shipped to the United States from Colombia. In France, Comps et al. (16) found rickettsialike organisms in the brain of moribund juvenile seabass (*Dicentrarchu labrax*) exhibiting abnormal swimming behavior and in Chile, Gaggero et al. (17) isolated *P. salmonis* from diseased coho salmon and rainbow trout held only in fresh water. A different, and as yet unidentified, bacterium has been isolated in fish cell cultures from diseased Atlantic salmon in Chile that had been reared only in fresh water.

Table 2. *Piscirickettsia salmonis* LF-89$^T$ observed in and/or isolated from fish

| Fish Host Species | Geo. Location | Salt Fresh Water | Obs./ Iso. | Ref. |
|---|---|---|---|---|
| Coho salmon (*Oncorhynchus kisutch*) | Chile | S[a] | I[c] | 6 |
| Rainbow trout (*Oncorhynchus mykiss*) | Chile | S | O/I[d] | 9 |
| Chinook salmon (*Oncorhynchus tshawytscha*) | | | | |
| Atlantic salmon (*Salmo salar*) | | | | |
| Chinook salmon | British Columbia Canada | S | O/I | 11 |
| Atlantic salmon | | | | |
| Coho salmon | | | | |
| Pink salmon (*Oncorhynchus gorbuscha*) | | | | |
| Atlantic salmon | Ireland | S | O[e] | 13 |
| Atlantic salmon | Norway | S | O/I | 12 |
| Coho salmon | Chile | F[b] | I | 18 |
| Rainbow trout | | | | |

[a] S = Host fish collected from salt water
[b] F = Host fish collected from fresh water
[c] I = Isolated in cell culture

The organism is smaller than *P. salmonis,* ca. 0.2-0.8 vs. ca. 0.5-1.5 in diameter, and both are usually coccoidal in shape. The unidentified bacterium was intracellular in spleen and kidney, did not grow on several types of bacteriologic media, and reportedly did not react with polyclonal antibodies against *P. salmonis* (18). The rickettsialike organisms observed in and/or isolated from nonsalmonid fish have not been characterized sufficiently to determine their relationship to *P. salmonis.* Taxonomic placement of these agents requires additional study.

*Neorickettsia helminthoeca*, the cause of the "salmon poisoning" disease of canids, is associated with fish but is not a fish pathogen. It is carried by the digenetic trematode, *Nanophyetus salmincola*, a parasite of salmonid fish in the Pacific Northwest (19). *N. helminthoeca* has been cultured in canine and murine cells but does not grow in cells of salmonid fish (20). Phylogenetically, *N. helminthoeca* is more closely related to the ehrlichiae and the rickettsia than it is to *P. salmonis* based on an analysis using the April 3, 1997, 16S rRNA gene sequence database containing 6,000 sequences. In addition, when *P.*

*salmonis* was tested with *N. helminthoeca* antiserum by indirect immunofluorescence, no reaction was observed (6).

There is no indication that *P. salmonis* or other rickettsial pathogens of fish cause disease in humans or other warm blooded animals. We speculate that the optimum temperature of 15°C to 18°C with no growth at 25°C and above prevents *P. salmonis* from becoming established in warm-blooded animals.

### *P. salmonis*

### Taxonomic Position

*P. salmonis* is the best characterized of the rickettsiae observed in and/or isolated from fish. The type species LF-89$^T$ was isolated from a diseased coho salmon from a sea water netpen site in southern Chile during an epizootic. The organism has been placed in a new genus in the order *Rickettsiales*, family *Rickettsiaceae* based primarily on their similar morphology and obligate intracellular nature (6). It has been deposited with the American Type Culture Collection as ATCC VR 1361.

Many rickettsiae observed in or isolated from fish since 1989 have been identified serologically as *P. salmonis* (Table 2). We tested isolates from coho salmon and rainbow trout from Chile and from Atlantic salmon reared in Chile; British Columbia, Canada; and Norway. All five isolates are morphologically similar to LF-89$^T$ and react with polyclonal antibodies against the type strain. Certain monoclonal antibodies developed in our laboratory can differentiate between these isolates (unpub. data). The 16S ribosomal DNA of these five isolates from three geographic locations were amplified by PCR to assess the genetic variability in this species or species-complex. The PCR products were sequenced and compared with other bacterial small subunit rRNA sequences. The genus Piscirickettsia belongs within the gamma subdivision of the proteobacteria and is not closely related to species of Erlichiae, Rickettsia, or Neorickettsia from the subdivision of alpha proteobacteria (Figure 1). The similarities

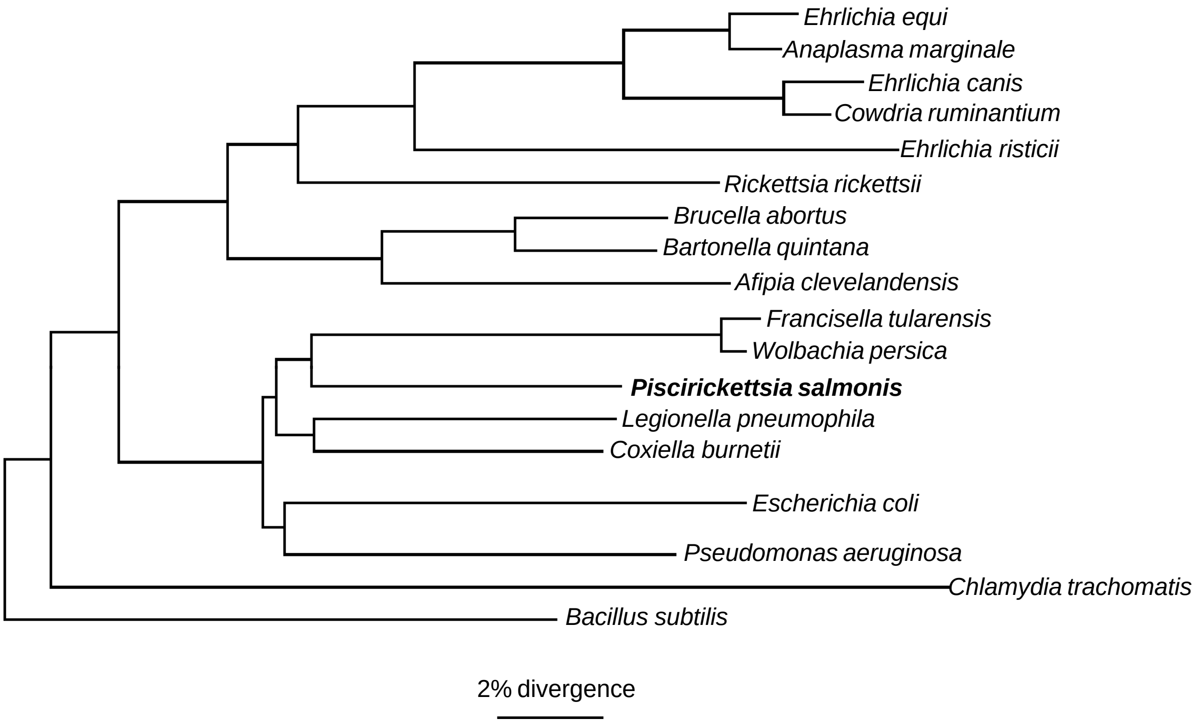

Figure 1. Phylogenetic relationships of *Piscirickettsia salmonis*, selected rickettsiae and bacteria. Evolutionary distances were calculated by the method of Jukes and Cantor (34). After eliminating regions of ambiguity and uncertain homology 1,313 positions of the 16S rDNA gene were compared.

between Piscirickettsia, Ehrlichiae, and Rickettsia species are approximately 80%. Similarities between Piscirickettsia, Coxiella, and Wolbachia are approximately 86% to 88%. The five *P. salmonis* isolates form a tight monophyletic cluster with relatedness of 99.7% to 98.5%. Two of the isolates from Chile and those from Norwegian and Canadian fish are closely related (>99.4% similarity). One isolate from Chile, EM-90, differed from the other four isolates (21). Similarity values for EM-90 were 98.5% to 98.9%.

To further clarify the genetic variability in this genus, the internal transcribed spacer (ITS) and 23S rDNA of six isolates have been analyzed. One spacer sequence was identified per isolate, and the region did not contain a tRNA gene. The ITS sequences were 311-bp in length and varied among the isolates (95.2% to 99.7% similarity). Only one ITS sequence was obtained for each of the *P. salmonis* isolates, suggesting the presence of one rRNA operon, which agrees with reports for other slow growing organisms (22).

Approximately 1,900-bp of the 23S rDNA gene have been analyzed for the six isolates, and similarities ranged from 97.9% to 99.8%. Three Chilean isolates and the Norwegian and Canadian isolates are closely related (99.1% to 99.7% ITS and 99.3% to 99.8% 23S rDNA similarities). The sequence of isolate EM-90 differed from those of the other five isolates (similarities ranged from 95.2% to 96.9% ITS and 97.6% to 98.5% 23S rDNA).

Phylogenetic trees constructed with the 16S, ITS, and 23S rDNA data showed similar topography, further reinforcing the relationships indicated between the isolates. Comparison of the *P. salmonis* ITS with the 16S rDNA region shows that it has diverged on average 3.15 times faster than the 16S rDNA gene, while the 23S rDNA gene has diverged 1.6 times faster than the 16S rDNA gene. This is similar to findings with other obligate intracellular bacteria (23).

### Characteristics of the Type Strain LF-89$^T$

*P. salmonis*, type strain LF-89$^T$ is a nonmotile, gram-negative, obligatedly intracellular bacterium. It is predominately coccoid (ca. 0.5-1.5 µm in diameter) and also occurs as rings or pairs of curved rods (Figure 2). It replicates within membrane-bound cytoplasmic vacuoles (Figure 3) in selected fish cells lines and in the cells of tissues throughout infected fish. Thin sections of the bacterium examined by electron microscopy display typical gram-negative cell walls and the protoplasmic structure of a prokaryote. Giemsa stains the rickettsiae cells dark blue. LF-89$^T$ does not react with the monoclonal antibody made against the group-specific chlamydial LPS antigen (5).

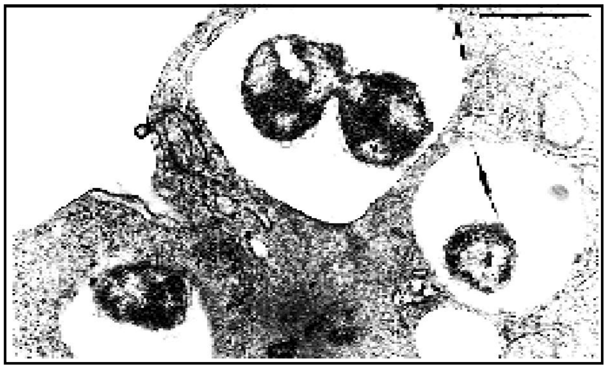

Figure 2. *Piscirickettsia salmonis* within a cytoplasmic vacuole in CHSE-214 cell line 4 days post inoculation. Note organisms dividing within vacuole. May Greenwald-Giemsa stain. Bar = 1 µm.

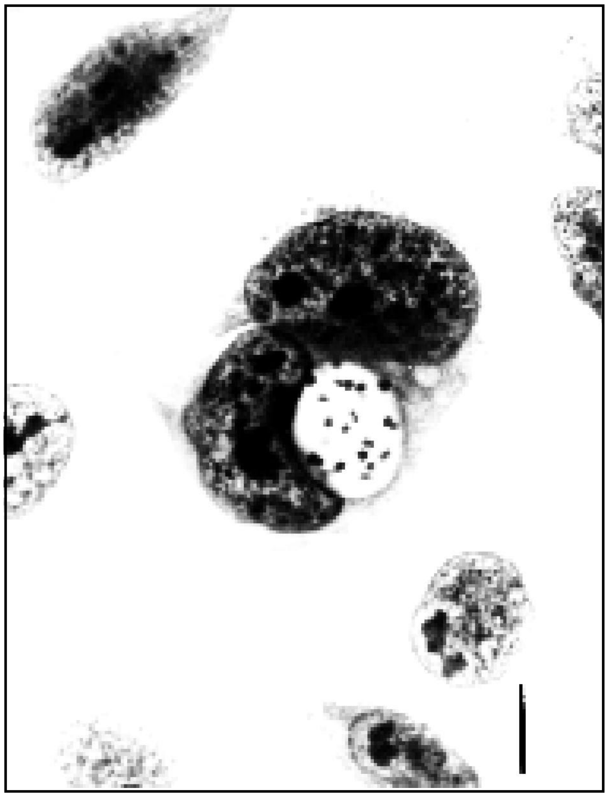

Figure 3. *Piscirickettsia salmonis* undergoing apparent binary fission within a vacuole in the cytoplasm of infected CHSE-214 cells. Bar = 10 µm.

*P. salmonis* can be cultivated in certain fish cell lines where it produces a cytopathic effect. It does not replicate on any known cell-free media, and its in vitro growth characteristics have been described (6). Replication is optimal at 15°C to 18°C, retarded above 20°C and below 10°C, and does not occur above 25°C. It replicates to titers of $10^6$ to $10^7$ $TCID_{50}$ $ml^{-1}$ in fish cell cultures. Titers are decreased 99% or more by a single cycle of freeze-thaw at -70°C. The addition of 10% DMSO to the freezing medium acts as a cryopreservative. In vitro, it is sensitive to a broad range of antibiotics.

Tenfold dilutions of spent medium from an LF-$89^T$infected cell culture injected into groups of 40 juvenile coho salmon and groups of 30 juvenile Atlantic salmon resulted in death rates of 88% to 100% during a 42-day experiment (8). Typical signs of the disease were present in the inoculated experimental coho, whereas in Atlantic salmon, the only gross sign of disease was death. The $LD_{50}$ was not obtained, but death in both species followed a dose-response pattern, and LF-$89^T$ was reisolated from each group inoculated.

Extracellular survival of LF-$89^T$ was tested under selected environmental conditions (24); infectivity remained for at least 14 days in preparations of semipurified LF-$89^T$ suspended in high salinity sea water at 5°C, 10°C, and 15°C. Infectivity was rapidly reduced in preparations suspended in fresh water. Titers dropped below the level of detection ($10^2$ $TCID_{50}$ $ml^{-1}$) immediately after suspension in fresh water, and no infectious material could be recovered from these preparations.

### Piscirickettsiosis

*P. salmonis* produces an epizootic disease of fish called piscirickettsiosis. Death rates associated with piscirickettsiosis in salmonids range from a high of 90% among coho in Chile (25) to a low of 0.06% in Canada and Norway (9,11). All species of salmonids cultured in Chile are affected by this disease, but the highest death rates occur in coho salmon cultured in salt water. Fish in sea water netpens begin to die 6 to 12 weeks after their transfer from fresh water (5). Deaths peak in the fall and rise again the following spring (26).

A variety of clinical signs are associated with *P. salmonis* infection, but few are specific to piscirickettsiosis. Moribund fish collect at the water surface along the edges of the sea cages; they are lethargic, dark, and show loss of appetite (9); the gills are pale, and hematocrits are frequently 25% or less. The first signs observed are often hemorrhages and lesions of the skin. The lesions range from small areas to shallow ulcers up to 2 cm in diameter (25). Internally, the kidney is swollen and the spleen enlarged. Petechial hemorrhages are found on the swim bladder and viscera. Diagnostic ringshaped, cream-colored lesions are present on the livers of chronically infected fish (Figure 4). In acute cases, death may be the only gross sign of disease (27).

The histopathologic symptoms associated with *P. salmonis* infection and the disease it causes have been described, but a great deal of work remains to be done (25-27). Piscirickettsiosis produces marked pathologic changes in most internal organs of infected fish, where severe changes occur in the intestine, kidney, liver, and spleen. Necrosis and inflammation may occur throughout the body, especially in cells adjacent to blood vessels. Epithelial hyperplasia results in lamellar fusion of the gills. The bacterium is commonly observed within macrophages and in the cytoplasm of infected host cells.

### Mode of Transmission

The source, reservoir, and means of transmission of *P. salmonis* continue to be important

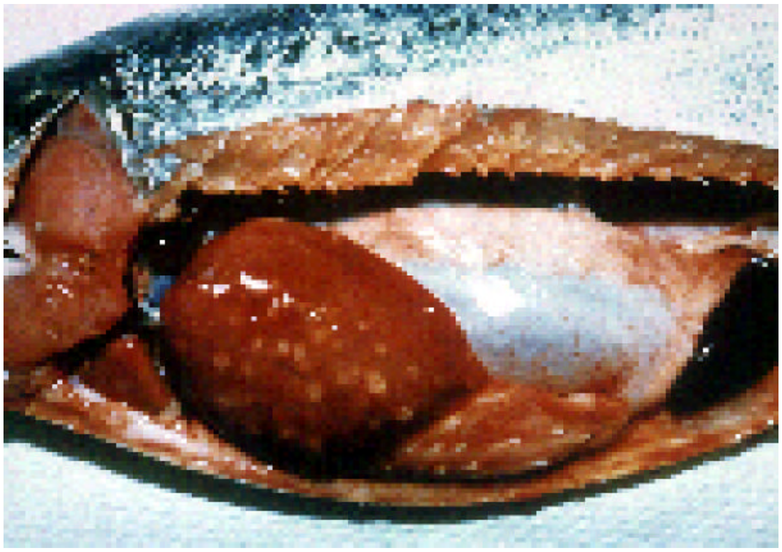

Figure 4. Coho salmon infected with *Piscirickettsia salmonis*. Note cream-colored lesions on liver, enlarged spleen, pale gills, and hemorrhaged areas within the peritoneal cavity.

areas of study. Although piscirickettsiosis has been observed in salmonid fish over a widespread geographic area (Table 2), with the exception of the predictable annual epizootics in Chile, the infections seem sporadic and of limited virulence in other parts of the world. No association other than the marine environment and the host species is apparent between locations in Canada, Norway, and Ireland. One or more naturally occurring aquatic animals, perhaps only transiently present, may provide the source and reservoir of these rickettsiae in the widely separated diverse areas. No alternate host has been identified.

*P. salmonis* has been experimentally passed by injection (8,26), but the normal mode of transmission for this agent (horizontal, vertical, or through a vector) has not been demonstrated. Except in *Coxiella burnetii*, an intermediate host or vector is required for transmission of rickettsiae in the terrestrial environment (28). *C. burnetii* not only has an intermediate host but appears to develop a sporogenic phase that protects it from drying when it is host or vector free. No vector or sporogenic phase has been observed for *P. salmonis*. *P. salmonis* may not require an intermediate host or sporogenic stage for survival and transmission in the aquatic environment. The extended extracellular survival time of this organism in salt water (24) may be of sufficient duration to permit horizontal transmission without a vector. The almost immediate deactivation of the rickettsia in fresh water makes direct horizontal transmission unlikely unless *P. salmonis* was protected by host(s) cell membranes or tissue exudates.

Limited research designed to demonstrate the role of horizontal transmission has provided mixed results. Garcés et al. (8) saw no evidence of horizontal transmission between injected coho salmon dying of piscirickettsiosis and uninjected salmon held in a cage in the same tank of flowing fresh water. Cvitanich et al. (26) reported horizontal transmission between injected and sham-injected coho salmon held in static fresh and sea water aquaria. Environmental conditions differed between experiments (e.g., flowing water at a mean temperature of 10.5°C vs. static water at 15°C), therefore, meaningful comparisons could not be made. Nevertheless, this research suggests that under certain circumstances, horizontal transmission is possible.

Although rickettsiae are found in the gonads of infected fish, vertical transmission of *P. salmonis* has not been demonstrated. The limited number of piscirickettsiosis cases reported in fresh water indicate that vertical transmission is, at best, rare. Until definitive studies are conducted, the questions of source, reservoir, and normal mode of transmission of *P. salmonis* remain unanswered.

### Detection and Identification

Inoculation of susceptible fish cell lines is the most sensitive method for detecting *P. salmonis* (29). However, isolation of an infectious agent sensitive to low levels of antibiotics routinely used in cell culture presents a problem. All cultures must be maintained in antibiotic-free medium.

Diagnostic specimens collected aseptically in the field may become contaminated by other bacteria. For this reason, preliminary diagnosis of piscirickettsiosis is normally made by examining Gram, Giemsa, methylene blue, or acridine orange-stained kidney imprints or smears, and confirming their identity by serologic methods, e.g., immunofluorescence (13) or immunohistochemistry (30).

A nested polymerase chain reaction (PCR) using universal 16S rDNA bacterial outer primers and *P. salmonis*-specific internal primers was developed to detect the genomic DNA. The nested PCR assay allowed detection of less than one *P. salmonis* tissue culture infectious dose 50 ($TCID_{50}$). Using the *P. salmonis*-specific primers in a single amplification allowed detection of 60 $TCID_{50}$. The specificity of PCR was assessed with a panel of four salmonid and 15 bacterial genomic DNA preparations. Products derived from amplification were observed only from *P. salmonis* DNA (31).

Restriction fragment length polymorphism analysis of the 16S rDNA products from six isolates of *P. salmonis* demonstrated that one isolate, EM-90, was different. Two additional primers were developed that differentiate EM-90 from the other five *P. salmonis* isolates (31).

PCR using DNA extracted from spleens of tilapia or paraffin-embedded tissue of the blue-eyed plecostomus did not produce amplification products. These tissues were collected and examined from fish during an epizootic caused by rickettsialike organism (unpub. data). Amplification using 16S rDNA universal primers was successful, which suggests that the rickettsialike organism infecting these fish were not *P. salmonis*.

### Control of the Disease

Although *P. salmonis* is sensitive in vitro to many of the antibiotics commonly used to control other infectious diseases of fish, e.g., tetracycline, erythromycin, and oxolinic acid (6,26), these preparations have not been useful in treating fish with piscirickettsiosis. Antibiotic levels may not reach sufficient concentrations within the host cells in vivo, to terminate replication of the pathogen.

The lack of effective methods for treating piscirickettsiosis has encouraged the development of disease prevention techniques. Vaccines have been successfully used for control of certain gram-negative bacterial diseases of fish; however, at present no efficacious preparations have been developed to protect fish against *P. salmonis*.

Questions remain concerning *P. salmonis* and the disease it causes in fish. The reservoir(s) of the infectious agent should be determined if the spread of the disease is to be controlled (32). There is a need to determine the mode(s) of transmission of *P. salmonis* to fully understand the pathogenesis of piscirickettsiosis. The infectivity of *P. salmonis* for native, nonsalmonid fish has not been investigated. Difference in virulence between *P. salmonis* in Chile and salmonids in the Northern Hemisphere is an important consideration. It must be determined if intrinsic differences in the isolates, the host fish, the environment, or some combination of factors is responsible for these differences. The rickettsiae from salmonid fish in the fresh water and marine environments should be compared, and the relationships between the rickettsiae from salmonid fish and those from other fish species should be determined.

A new group of fish pathogens was recognized in 1989 with the isolation and identification of *P. salmonis* LF-89$^T$ from cultured salmonids in Chile (5,6). The rickettsial etiology of an infectious disease of fish in a variety of locations and host species suggests that they are an important group of emerging fish pathogens (33). These pathogens have caused severe mortality among cultured salmonids in Chile and are not present among stocks of important food fish cultured in British Columbia, Canada, Norway, and Ireland. Taiwan has experienced serious losses amon talapia, another important food fish. At least one species imported for aquaria are also infected with a rickettsialike agent. The lack of antimicrobial drugs and vaccines for prevention and control emphasize the need for additional research. The method of transmission of *P salmonis* is not understood and needs to be researched. The movement of fish and spread of the agent make the develoment of improved diagnostic techniques important.

### Acknowledgments

We thank Dr. Sandra Bravo of Puerto Montt, Chile, for the photograph of a diseased fish in Figure 4. Preparation of this paper was supported in part by the Oregon Sea Grant with funds from the National Oceanic and Atmospheric Administration Office of Sea Grant, Department of Commerce, under grant NA36RG0451, project R/FSD-22. This is Oregon Agricultural Experiment Station Technical Paper Number 11,090.

Dr. Fryer has been a faculty member of the Department of Microbiology at Oregon State University (OSU) for more than 35 years, serving as chair for 20 of those years. His research has focused primarily on the infectious diseases of Pacific salmon and other species of fish. He is professor emeritus and director of the Center for Salmon Disease Research at OSU.

Dr. Mauel received his degree from the Department of Microbiology at Oregon State University in 1996 and is a postdoctoral fellow at the Center for Vector-borne Disease, University of Rhode Island. His scientific interests concern the molecular biology of fastidious intracellular pathogenic bacteria.

### References

1. Mohamed Z. The discovery of a rickettsia in a fish. Ministry Agriculture Cairo, Technical Science Service, Veterinary Section Bulletin 1939;214:1-6.
2. Ozel M, Schwanz-Pfitzner I. Vergleichende elektronenmikroskopische Untersuchungen an Rhabdoviren pflanzlicher und tierischer Herkunft: III. Egtved-Virus (VHS) der Regenbogenforelle (*Salmo gairdneri*) und Rickettsienahnliche Organismen. Zentralblatt fuerr Bakteriologie Mikrobiologie und Hygiene, I Abt Originale A 1975;230:1-14.
3. Wolf K, Quimby MC. Established eurythermic line of fish cells *in vitro*. Science 1962;135:1065-66.
4. Davies AJ. A rickettsia-like organism from dragonets, *Callionymus lyra* L. (Teleostei: Callionymidae) in Wales. Bulletin of the European Association of Fish Pathologists 1986;6:103.
5. Fryer JL, Lannan CN, Garcès LH, Larenas JJ, Smith PA. Isolation of a rickettsiales-like organism from diseased coho salmon *Oncorhynchus kisutch* in Chile. Fish Pathology 1990;25(2):107-14.

6. Fryer JL, Lannan CN, Giovannoni SJ, Wood ND. *Piscirickettsia salmonis* gen. nov., sp. nov., the causative agent of an epizootic disease in salmonid fishes. Int J Syst Bacteriol 1992;42:120-6.
7. Lannan CN, Winton JR, Fryer JL. Fish cell lines: establishment and characterization of nine cell lines from salmonids. In Vitro 1984;20:671-6.
8. Garcés LH, Larenas JJ, Smith PA, Sandino S, Lannan CN, Fryer JL. Infectivity of a rickettsia isolated from coho salmon (*Oncorhynchus kisutch*). Diseases of Aquatic Organisms 1991;11:93-7.
9. Brocklebank JR, Speare DJ, Armstrong RD, Evelyn T. Septicemia suspected to be caused by a rickettsia-like agent in farmed Atlantic salmon. Can Vet J 1992;33:407-8.
10. Evelyn TPT. Salmonid rickettsial septicemia. In: Kent ML, editor) Diseases of seawater netpen-reared salmonid fishes in the Pacific Northwest. Canadian special Publication Fish Aquatic Science 116. Nanaimo (BC): Department Fisheries and Oceans 1992;18-9.
11. Olsen AB, Evensen Ø, Speilberg L, Melby HP, Håstein T. <<Ny>> laksesykdom forårsaket av rickettsie. Norsk Fiskeoppdrett NR 1993;12:40-1.
12. Rodger HD, Drinan EM. Observation of a rickettsia-like organism in Atlantic salmon, *Salmo salar* L., in Ireland. J Fish Dis 1993;16:361-9.
13. Lannan CN, Ewing SA, Fryer JL. A fluorescent antibody test for detection of the rickettsia causing disease in Chilean salmonids. Journal of Aquat Animal Health 1991;3:229-34.
14. Chern RS, Chao CB. Outbreaks of a disease caused by a rickettsia-like organism in cultured tilapias in Taiwan. Fish Pathology 1994;29:61-71.
15. Khoo L, Dennis PM, Lewbart GA. Rickettsia-like organisms in the blue-eyed plecostomus, *Panaque suttoni* (Eigenmann & Eigenmann). Journal of Fish Diseases 1995;18:157-64.
16. Comps M, Raymond JC, Plassiart GN. Rickettsia-like organism infecting juvenile sea-bass *Dicentrarchus labrax*. Bulletin of the European Association of Fish Pathologists 1996;16:30-3.
17. Gaggero A, Castro H, Sandino AM. First isolation of *Piscirickettsia salmonis* from coho salmon, *Oncorhynchus kisutch* (Walbaum), and rainbow trout, *Oncorhynchus mykiss* (Walbaum), during the freshwater stage of their life cycle. Journal of Fish Diseases 1995;18:277-9.
18. Cvitanich JD, Garate NO, Silva PC, Andrade VM, Figueroa PC, Smith CE. Isolation of a new rickettsia-like organism from Atlantic salmon in Chile. AFS/FHS Newsletter 1995;23:1-3.
19. Milleman RE, Knapp SE. Biology of *Nanophyetus salmincola* and "salmon poisoning" disease. Adv Parasitol 1970;8:1-41.
20. Noonan WE. *Neorickettsia helminthoeca* in cell culture (dissertation). Corvallis (OR): Oregon State University, 1973.
21. Mauel MJ. Evidence for molecular diversity of Piscirickettsia salmonis (dissertation). Corvallis (OR): Oregon State University, 1996.
22. Frothingham R, Wilson KH. Sequence-based differentiation of strains in the *Mycobacterium avium* complex. J Bacteriol 1993;175:2818-25.
23. Stothard DR, Clark JB, Furst PA. Ancestral divergence of *Rickettsia bellii* from the spotted fever and typhus groups of *Rickettsia* and antiquity of the genus *Rickettsia*. Int J Syst Bacteriol 1994;44:798-804.
24. Lannan CN, Fryer JL. Extracellular survival of *Piscirickettsia salmonis*. Journal of Fish Diseases 1994;17:545-8.
25. Branson EJ, Nieto Diaz-Munoz D. Description of a new disease condition occurring in farmed coho salmon, *Oncorhynchus kisutch* (Walbaum), in South America. Journal of Fish Diseases 1991;14:147-56.
26. Cvitanich JD, Garate NO, Smith CE. The isolation of a rickettsia-like organism causing disease and mortality in Chilean salmonids and its confirmation by Koch's postulate. Journal of Fish Diseases 1991;14:121-45.
27. Larenas HJ, Hidalgo VL, Garcés AH, Fryer JL, Smith SP. Piscirickettsiosis: lesiones en salmón del Atlántico (*Salmo salar*) infectados naturalmente con *Piscirickettsia salmonis*. Avances en Ciencias Veterinarias 1995;10:53-8.
28. Weiss E, Moulder JW. Order I. Rickettsiales Gieszczkiewicz 1939, 25[AL], In: Krieg NR, editor. Bergey's Manual of Systematic Bacteriology, Vol. 1. London: Williams and Wilkins, 1984:687-729.
29. Lannan CN, Fryer JL. Recommended methods for inspection of fish for the salmonid rickettsia. Bulletin of the European Association of Fish Pathologists 1991;11:135-6.
30. Alday-Sanz V, Rodger H, Turnbull T, Adams A, Richards RH. An immunohistochemical diagnostic test for rickettsial disease. Journal of Fish Diseases 1994;17:189-91.
31. Mauel MJ, Giovannoni SJ, Fryer JL. Development of polymerase chain reaction assays for detection, identification, and differentiation of *Piscirickettsia salmonis*. Diseases of Aquatic Organisms 1996;26:189-95.
32. Fryer JL, Lannan CN. Rickettsial and chlamydial infections of freshwater and marine fishes, bivalves, and crustaceans. Zoological Studies 1994;33:95-107. (Translated into Japanese by Professor Tokuo Sano and appears in Fish. Res. 1995; 14(3):54-65.)
33. Fryer JL, Lannan CN. Rickettsial infections of fish. Annual Review of Fish Diseases 1996. In press.
34. Jukes TH, Cantor CR. Evolution of protein molecules. In: Munro HN, editor. Mammalian protein metabolism. New York: Academic Press Inc., 1996:21-132.
